# Study of the Relationship between HPA-1 and HPA-5 Gene Polymorphisms and Refractory to Platelet Therapy and Recombinant Factor VII in Glanzmann Thrombasthenia Patients in Southeast of Iran

**Published:** 2018-01-01

**Authors:** Majid Naderi, Manizheh Habibpour, Shaban Alizadeh, Zahra Kashani Khatib, Akbar Dorgalaleh, Mohammed Awal Issah, Fatemeh Naadali

**Affiliations:** 1 Genetic Researcher Center in Non-Communicable Disease, Zahedan University of Medical Sciences, Zahedan, Iran; 2 Department of Hematology, School of Allied Medical Sciences, Tehran University of Medical Sciences, Tehran, Iran; 3 Department of Hematology, High Institute for Research and Education in Transfusion Medicine, Iranian Blood Transfusion Organization, Tehran, Iran; 4 Department of Hematology, School of Allied Medical Sciences, Iran University of Medical Sciences, Tehran, Iran; 5 Department of Hematology, International Campus, School of Allied Medical Sciences, Tehran University of Medical Sciences, Tehran, Iran

**Keywords:** Human platelet Ag-1, Human platelet Ag-5, Platelet therapy, Glanzmann thrombastenia

## Abstract

**Background:** Glanzmann Thrombasthenia (GT) is a rare autosomal disease. HPA (Human Platelet Alloantigen) is a surface polymorphic alloantigen of platelets. This study was intended to investigate and compare the polymorphism of HPA-1 and HPA-5 genes in two groups of GT patients, with and without resistance to platelet and recombinant factor VII therapy.

**Materials and Methods: **This case control study was performed on GT patients (n=16) with resistance to platelet therapy and recombinant factor VII and control group of GT patients (n=16) without resistance to platelet therapy and recombinant factor VII. The consent form was completed by each patient. Gene polymorphisms of HPA-1 and HPA-5 were investigated using SSP-PCR, and the obtained data were analyzed using statistical software SPSS16.0.

**Results:** The results indicated no significant relationship between the studied genes and their resistance to platelet therapy and recombinant factor VII. The frequencies of HPA-1 genotype a/a were 98% and 94% in patient and control groups, respectively. The frequency of allele b was found to be less than allele a. The value of this allele was 4% in patient group and 1% in control group. In addition, the HPA-5a/a (98%) was the most frequent alloantigen?? (check it) in both groups. Seven percent (7%) of the patients had the HPA-5a/b genotype, and the HPA-5b/b was found to be absent in these individuals.

**Conclusion:** According to the results obtained, it could be concluded that these genes play no role in resistance to platelet therapy.

## Introduction

 Glanzmann Thrombasthenia (GT) is a rare autosomal disease, caused by reduction or defects in the activity of membrane αIIbβ3 integrin receptor or Glycoprotein (GP) complex IIb / IIIa with lack of platelet aggregation. This GP is an abundant platelet GP that plays a key role in coagulation. Both genes are located on the long arm of chromosome 17(17q21-q23)(1-5). GT is a mild to severe bleeding disorder in the mucosal surfaces. The clinical symptoms of the homozygous patients are petechiae, purpura, ecchymosis, menorrhagia, epistaxis, gingival bleeding, etc; however, it is often possible that the heterozygous patients shows no clinical symptoms ^3^^-^^4^^, ^^6^ . 

GT is classified into three types based on the expressed platelet GPIIb/ IIIa levels^   2 ^.

Therapy involves both preventive measures and treatment of specific bleeding episodes. Platelet transfusion and recombinant factor VII are important and standard treatments. But many patients show alloimmunization, especially after several platelet transfusions, which can cause serious side effects such as excessive bleeding during surgery, delivery or even tooth extraction without taking Precautions^3^^,^^12^. By finding the relationship between some alloantigens like HPA-1, HPA-5 gene polymorphisms and therapy refractory, patients can be properly cured by using platelets matched via HPA-1,5 without the occurrence of alloimmunizations.

 HPA (Human Platelet Alloantigen) is a surface polymorphic alloantigen of platelets. Alloantibody is produced in patients who frequently receive platelet and it leads to resistance of treatment. HPA-1 is the most important platelet alloantigen system that is expressed in GPIIIa and the antibody against HPA-1 is commonly used in clinical laboratories^3^.

HPA-5 is expressed in GPIa. There are a few studies on GT in Iran. The prevalence of this disorder is significant in southeast of Iran because of the high rate of consanguineous marriages^1^. This study was intended to investigate and compare the polymorphisms of the HPA-1 and HPA-5 genes in two groups of GT patients, those with and without resistance to platelet therapy and recombinant factor VII.

## MATERIAL AND METHODS

**Study population**

The case control study was performed during 2014 to 2015 at Imam Khomeini hospital in Sistan & Balouchestan province, southeast of Iran. They were 16 GT patients who were resistant to platelet therapy and recombinant factor VII and the control group consisted of 16 GT patients without resistance to platelet therapy and recombinant factor VII. The consent form was completed by each patient. All demographic data, including age, parental consanguinity, clinical manifestations and family history were extracted from patients´ records. Each patient in control group was matched with case group.

**DNA Extraction**

Genomic DNA was extracted from each patient’s blood using DNA extraction kit (Viogene). The quality of obtained DNA was evaluated by agarose gel electrophoresis containing 0.5 g/ml Ethidium bromide. 

**Polymerase chain reaction (PCR)**

**Figure 1:**
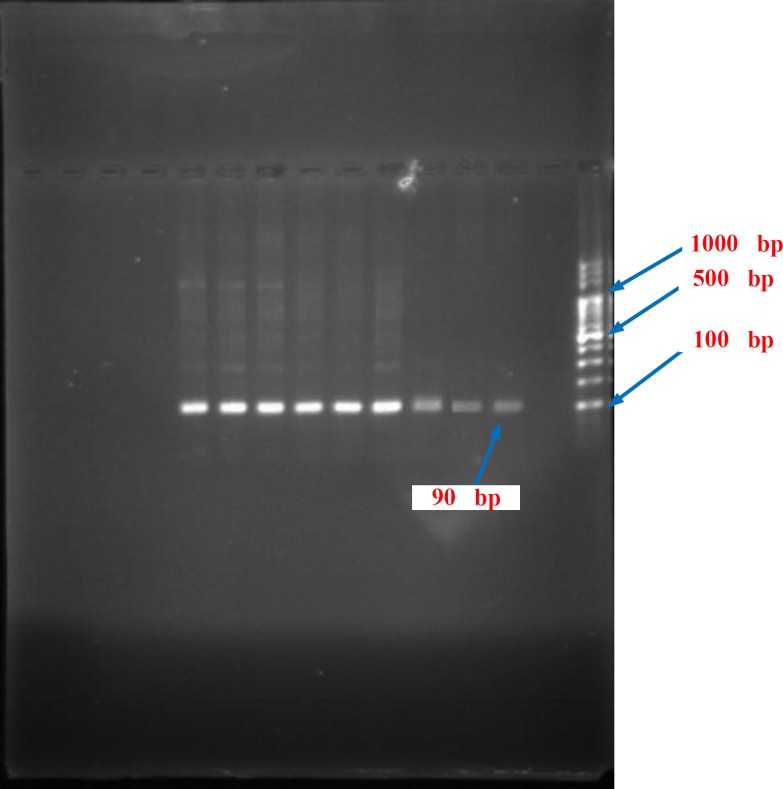
The PCR products of the HPA-1 gene of patients by DNA Ladder 100 bp on the agar gel

**Figure 2:**
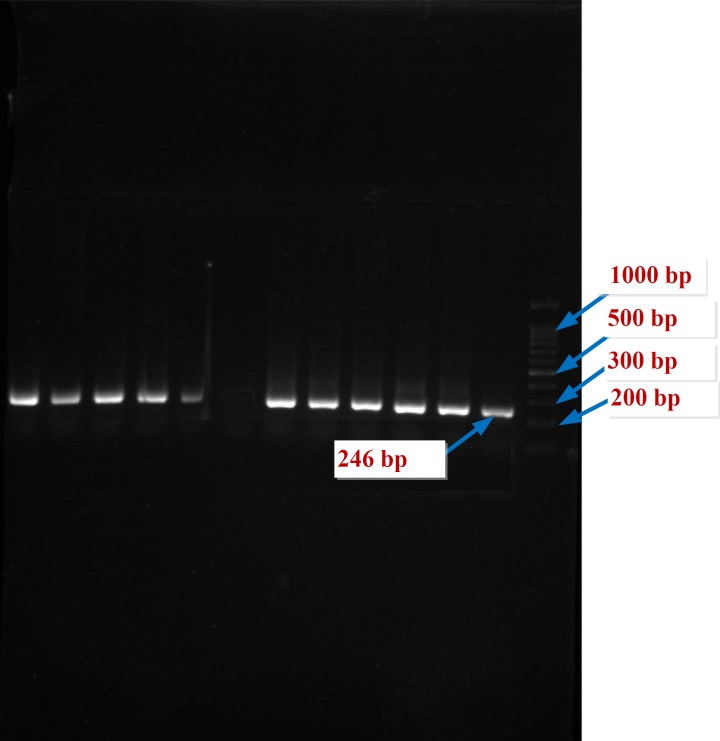
The PCR products of the HPA-5 gene of controls by DNA Ladder 100 bp on the agar gel

**Figure 3:**
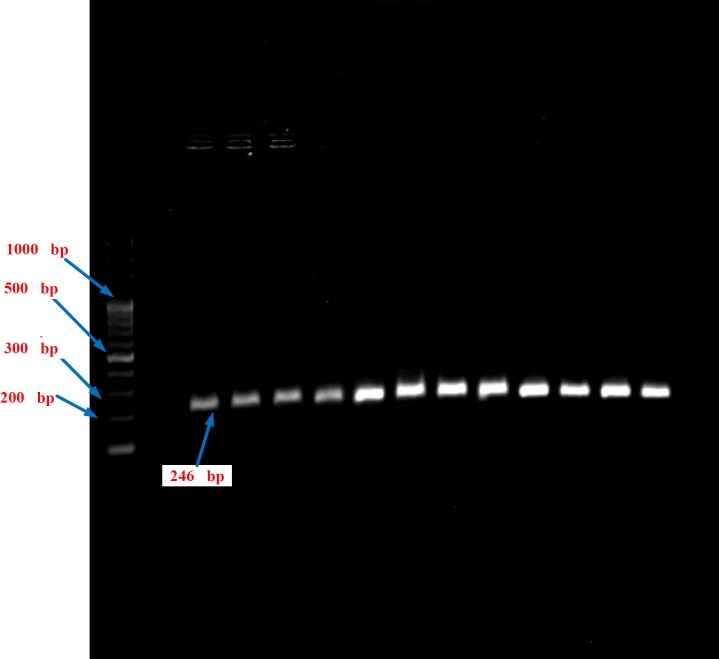
The PCR products of the HPA-5 gene of patients by DNA Ladder 100 bp on the agar gel

**Figure 4:**
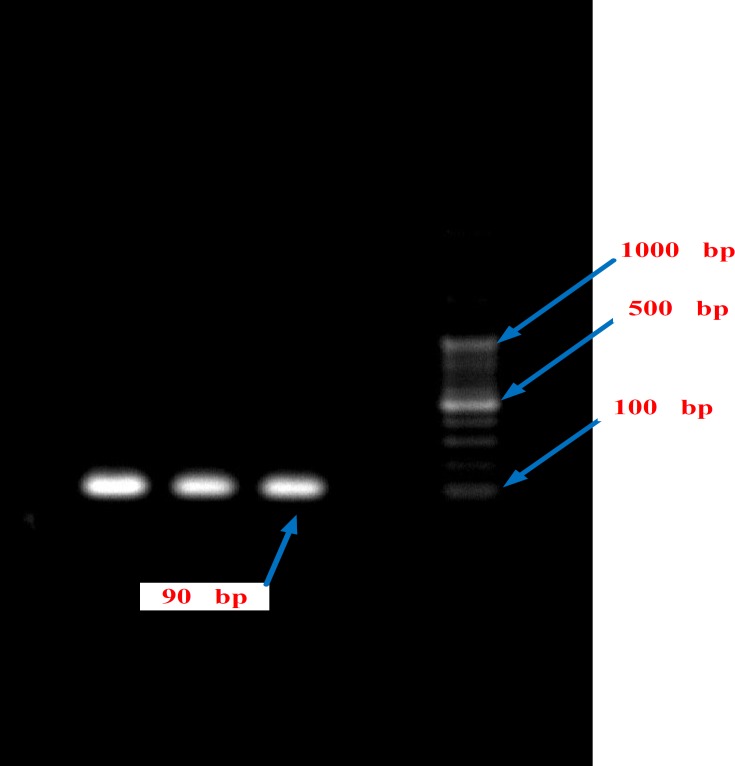
The PCR products of the HPA-1 gene of controls by DNA Ladder 100 bp on the agar gel

The primers of HPA-1 and HPA-5 gene polymorphisms are shown in Table1. PCR procedure was performed under standard conditions as shown in Table 2. The products were then separated on an agar gel Electrophoresis. The images of the gene’s PCR products are shown in Figures 1-4. 

**Table 1:** Primers for HPA-1 and HPA-5 gene polymorphisms

position	primer sequences	Base pair
**HPA-1**	1a: TCACAGCGAGGTGAGGCCA 1b: TCACAGCGAGGTGAGGCCG Common primer: GGAGGTAGAGAGTCGCCATAG	90
**HPA-5**	5a: AGTCTACCTGTTTACTATCAAAG 5b: AGTCTACCTGTTTACTATCAAAA Common primer: CTCTCATGGAAAATGGCAGTG	246

**Table 2:** PCR condition for HPA-1 and HPA-5 gene polymorphisms

**Cycles**		**Temperature**	**Time**
First Denaturing		_94_^ 0^C	5 min
	Denaturing	^ 0^C	sec
30 Cycles	Annealing	^0^C	Sec
	Extension	^0^C	30 Sec
Final Extension		^0^C	5 min

**PCR-Sequencing**

 The PCR products after purification were sent to Pishgam Company for sequencing, and then it was analyzed by the Chromas software. 

**Statistical analysis**

All obtained results were reported as mean ± standard deviation (SD) and percentages. HPA-1 and HPA-5 gene polymorphisms were finally compared in case and control groups.

## Results

**Figure 5:**
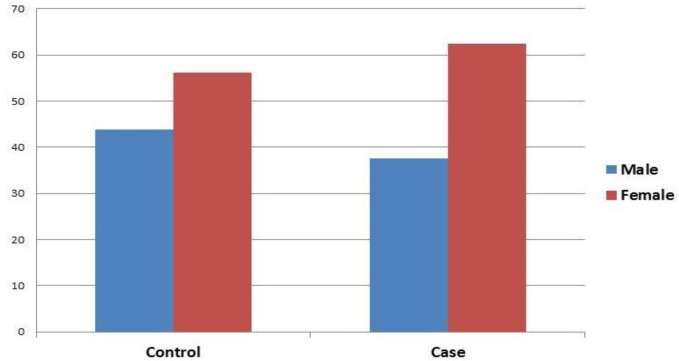
Distribution of sex frequency of studied individuals in patients with resistance to platelet treatment and recombinant factor VII and the control group

 The age and sex of Glanzmann Thrombasthenia patients with resistance to treatment were matched with the control group. All patients were from a same geographical area to avoid environmental impacts. The average age of participants was 22.066 (standard deviation=7.66). The mean age of Glanzmann Thrombasthenia patients with resistance to platelet therapy and recombinant factor VII was 20.62 years and within the range of 9-32 years old; but the mean age of control group was 21.5 years and within the range of 3-39 years. The sex frequency distribution of the subjects in the control and patient groups is shown in Figure 5. 

**Genotype and allele frequencies**

The Genotype and allele frequencies of the HPA gene were performed using PCR method and sequencing. The results of genotype and allele frequencies are shown in Table 3. It is clear that the genotype frequency of HPA-1a/a in both groups of patients and controls is high. In addition, although the frequency of allele HPA-1 b in patients is high, there is no statistically significant difference. Fig 3 also clarifies the Genotype frequencies of HPA-5a/a among Glanzmann Thrombasthenia patients. Frequency percentages were 93.5% and 100% for treatment-resistant patients and the control group, respectively. 

**Table 3:** The Genotype and allele frequency of the HPA-1 and HPA-5 in patients and control group

HPA type	Genotype and Allele frequency	Both group N=32	Control	Refractory patient
HPA-1	1a/a	96%	98%	94%
	1a/b	4%	2%	6%
	1a	0.98	0.99	0.96
	1b	0.02	0.01	0.04
HPA-5	5a/a	96.5%	100%	0.93%
	5a/b	3.5%	0	7%
	5a	0.99	1.0	0.97
	5b	0.01	0.0	0.03

## Discussion

 In the present work, the frequency of the polymorphisms HPA-1 and HPA-5 in Glanzmann Thrombasthenia patients and the control group were examined and compared. This study was conducted on a total of 16 patients and 16 controls. The frequencies of HPA-1 genotype a/a were 98% and 94% in the patient and control group, respectively. These results are in accordance with the results of Madani et al. study, which involved the use of blood samples of blood donors. Also, HPA-1b/b genotype was not reported in the above-mentioned study, which also agrees with our study’s results. Furthermore, the results of the genotypes of HPA-1a/b (96:4) obtained in the present work are comparable with the results of other studies conducted in Saudi Arabia (100: 0), China (99.4:0.6), Korea (99:1) and India (100:0)^   7 ^. The result of this study is also consistent with the result of Tan (2012), which was conducted in Malaysia. In his study, the frequent HPA was reported to be the variant HPA-1a/a, and allele b was found to be of low frequency which are similar to the findings of our study^   5 ^. The frequency of allele b was found to be less than allele a. The value of this allele was 4% in patients and 1% in control group. This value is higher for patients than the range of Asian Allele b, but it was not statistically significant. Although this result is high compared with the Asian value, it is however low compared with the amount of allele b among the European population. It should be noted that the present study was a comparative study conducted on healthy and patient groups, while the other studies were conducted on only healthy people. In this current study, the significant relationship between polymorphisms HPA-1 and resistance to platelet therapy and recombinant factor VII were not found in both patients and healthy Individuals which is in agreement with Tanboga (2013) study that was conducted on coronary artery patients. No relationship was observed by Tanboğa on angiography between patients receiving clopidogrel and polymorphisms HPA-1. Therefore, they concluded that HPA-1 gene polymorphism is not associated with platelet aggregation or resistance to clopidogrel^   8 ^. However, the results of this study are not compatible with the results of Soundravally et al (2007). They conducted a study on patients with hemorrhagic fevers and detected no significant relationship between the HPA-1 heterozygous genotype and the incidence of hemorrhagic fevers. The heterozygous individuals are highly susceptible to the disease, while the people with genotype HPA1a/1b are highly at risk of dengue shock syndrome^   9 ^. The investigation of HPA-5 frequency showed the HPA-5a/a as the most frequent genotype in both study groups. It was observed in the entire control group, while 7% of the patients had the HPA-5a/b genotype and the HPA-5b/b was absent in the individuals. The frequency of the allele b was observed in 3% of the patient group, and it was not detected in the control group. Madani et al. also had a 98% frequency of HPA-5a/a in blood donors^   7 ^. Shayegan et al. conducted a study in Tehran in 2011. They found HPA-5 a/a (99%) as the most frequent HPA-5 genotype; it was seen in the control group and was 90.5% in the patient group. This confirms the finding of this present study. Shaygan et al. found HPA-5b frequency of 1% in both patient and control groups. However, in our study, the frequency of allele b in patient group was found to be high compared with the control group. Although 3% of the patients had the HPA-5b, this allele was not detected in the control group^   6 ^. In this present study, the genes of HPA-1 and HPA-5 were not seen in the homozygous allele b, which was consistent with Shayegan et al. study. Also, this has not been reported by other studies ^6^^, ^^10^^-^^11^ .

## CONCLUSION

 This study aimed to investigate the gene polymorphisms of HPA-1 and HPA-5 in Glanzmann Thrombasthenia patients with resistance to platelet therapy and recombinant factor VII compared with the control group in Southeast of Iran. It was observed that there was no relationship between the alleles, genotype of these two genes, and the resistance to platelet therapy and recombinant factor VII. This may point out to the possible involvement of other genes in resistance phenomenon, thus other alloantigen should be investigated. This is the first report in the context of resistance to platelet therapy in Glanzmann Thrombasthenia patients and the obtained data can be used as basic data for other studies since epidemiogenetic recognition is considered as one of the important principle in diagnostic and therapeutic planning. 
